# Functionally distinct high and low theta oscillations in the human hippocampus

**DOI:** 10.1038/s41467-020-15670-6

**Published:** 2020-05-18

**Authors:** Abhinav Goyal, Jonathan Miller, Salman E. Qasim, Andrew J. Watrous, Honghui Zhang, Joel M. Stein, Cory S. Inman, Robert E. Gross, Jon T. Willie, Bradley Lega, Jui-Jui Lin, Ashwini Sharan, Chengyuan Wu, Michael R. Sperling, Sameer A. Sheth, Guy M. McKhann, Elliot H. Smith, Catherine Schevon, Joshua Jacobs

**Affiliations:** 1 Mayo Clinic Medical Scientist Training Program, Mayo Clinic College of Medicine and Science, Mayo Clinic, Rochester, MN 55905 USA; 2 Department of Biomedical Engineering, Columbia University, New York, NY 10027 USA; 3 Department of Neurology, University of Texas, Austin, TX USA; 4 Department of Radiology, University of Pennsylvania, Philadelphia, PA 19104 USA; 5 Department of Neurosurgery, Emory University, Atlanta, GA 30322 USA; 6 Department of Neurological Surgery, University of Texas Southwestern, Dallas, TX 75390 USA; 7 Department of Neurological Surgery, Thomas Jefferson University, Philadelphia, PA 9107 USA; 8 Jefferson Comprehensive Epilepsy Center, Thomas Jefferson University, Philadelphia, PA USA; 9 Department of Neurology, Thomas Jefferson University, Philadelphia, PA 19107 USA; 10 Department of Neurological Surgery, Baylor College of Medicine, Houston, TX 77030 USA; 11 Department of Neurosurgery, Columbia University Medical Center, New York, NY 10032 USA; 12 Department of Neurosurgery, University of Utah, Salt Lake City, UT USA; 13 Department of Neurology, Columbia University Medical Center, New York, NY 10032 USA

**Keywords:** Learning and memory, Hippocampus

## Abstract

Based on rodent models, researchers have theorized that the hippocampus supports episodic memory and navigation via the theta oscillation, a ~4–10 Hz rhythm that coordinates brain-wide neural activity. However, recordings from humans have indicated that hippocampal theta oscillations are lower in frequency and less prevalent than in rodents, suggesting interspecies differences in theta’s function. To characterize human hippocampal theta, we examine the properties of theta oscillations throughout the anterior–posterior length of the hippocampus as neurosurgical subjects performed a virtual spatial navigation task. During virtual movement, we observe hippocampal oscillations at multiple frequencies from 2 to 14 Hz. The posterior hippocampus prominently displays oscillations at ~8-Hz and the precise frequency of these oscillations correlates with the speed of movement, implicating these signals in spatial navigation. We also observe slower ~3 Hz oscillations, but these signals are more prevalent in the anterior hippocampus and their frequency does not vary with movement speed. Our results converge with recent findings to suggest an updated view of human hippocampal electrophysiology. Rather than one hippocampal theta oscillation with a single general role, high- and low-frequency theta oscillations, respectively, may reflect spatial and non-spatial cognitive processes.

## Abstract (web summary)

We show that the human hippocampus exhibits two distinct theta oscillations during spatial navigation with the faster oscillation in posterior regions showing movement modulation. This result suggests a distinct feature of the human hippocampus compared to rodents, which generally show a single 8 Hz rhythm.

## Introduction

The theta oscillation is a large-scale network rhythm that appears at ~4–10 Hz in rodents and is hypothesized to play a fundamental role in mammalian spatial navigation and memory^1^. However, in humans, there is mixed evidence regarding the relevance and properties of hippocampal theta. Some studies in humans show hippocampal oscillations at 1–5 Hz that have similar functional properties as the theta oscillations seen in rodents^2–6^. There is also evidence that human movement-related hippocampal theta oscillations vary substantially in frequency according to whether a subject is in a physical or virtual environment^7–10^. Together, these studies and others have been interpreted to suggest that the human hippocampus does show a signal analogous to theta oscillations observed in rodents, but that this oscillation is more variable and slower in frequency^6^. These apparent discrepancies in the frequency of theta between species and behaviors shed doubt on the notion that theta exists as a single general oscillatory phenomenon that coordinates brain-wide neural activity consistently across species and tasks.

**Fig. 1:**
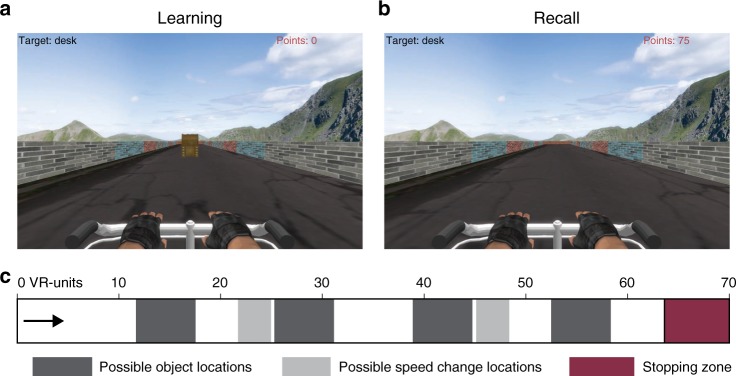
Spatial memory task. **a** Task screen image during a learning trial, where the object is visible as the subject travels down the track. **b** Task image during a recall trial, in which the object is invisible and the subject must recall the object location. **c** Task schematic, showing possible object and speed-change locations.

Our study aimed to resolve these discrepancies by characterizing the properties of human hippocampal oscillations in spatial cognition. We analyzed intracranial electroencephalographic (iEEG) recordings from the hippocampi of 14 neurosurgical subjects performing a virtual-reality (VR) spatial navigation task, in which subjects were asked to remember the location of an object as they were moved along a linear track (Fig. 1). A distinct feature of our experimental design compared with previous work is that our task randomly varied the subjects’ movement speed along the virtual track. This design encouraged subjects to continually attend to their spatial location throughout movement, because a nonspatial strategy based on remembering the time delay to each object would not be viable due to speed changes within trials. We hypothesized that this feature of our task would more clearly elicit human hippocampal oscillations specifically related to navigation. In addition, we recorded signals at various positions along the anterior–posterior (AP) axis of the hippocampus, including sites located considerably more posterior than those seen in previous work of this type, which allowed us to probe the anatomical organization of these oscillations.

Given the anatomical differences in the hippocampus between rodents and humans^11^, in this paper we test the hypothesis that understanding the spatial organization of human theta could help explain the apparent interspecies differences that have been reported previously. Here, we analyze the spectral and functional features of human hippocampal oscillations and test their consistency along the length of the hippocampus. In contrast to earlier work that generally emphasized a single theta oscillation for a given behavior, we instead find that the hippocampus showed multiple oscillations at distinct frequencies (often at ~3 Hz and ~8 Hz), even in a single subject. Further, ~8-Hz oscillations in the posterior (but not anterior) hippocampus often correlates with spatial processing. By demonstrating multiple patterns of hippocampal oscillations with different anatomical and functional properties, our findings suggest that human hippocampal oscillations at different frequencies are generated by separate anatomical networks to support distinct functions.

## Results

### Task description

**Fig. 2:**
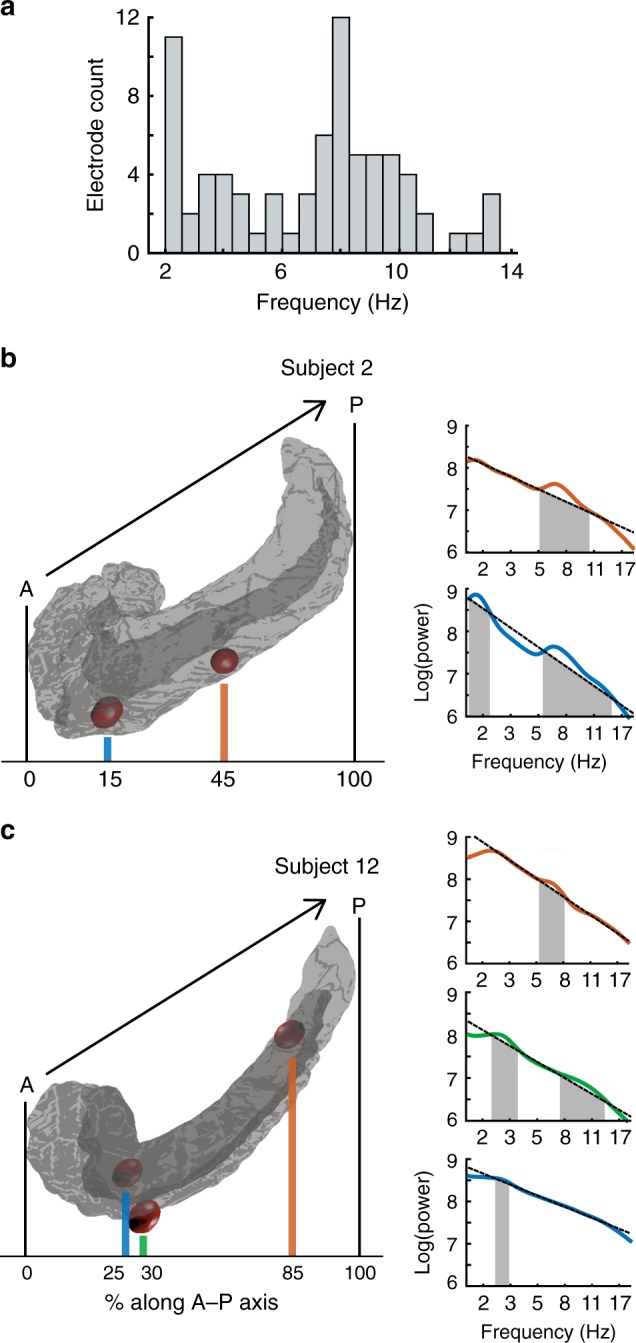
Power spectra of electrodes along the anterior–posterior axis of the hippocampus. **a** The distribution of detected oscillations across all hippocampal electrodes in our data set. **b** Rendering of Subject 2’s left hippocampus (left) and the power spectra (right) for electrodes implanted in this area. Shading in the power spectrum indicates detected narrowband oscillations. **c** Rendering of Subject 12’s left hippocampus and power spectra for the implanted electrodes.

Fourteen neurosurgical subjects (eight males and six females, age range 23–49) performed our virtual-reality (VR) spatial memory task, as we recorded neural activity from iEEG electrodes implanted in their hippocampi. The task^12^ required that subjects press a button to indicate when they were located at the position of a specified hidden object as they were moved at a randomly varying speed in one direction along a linear track (Fig. 1). Overall, subjects performed the task well, responding accurately (error distance ≤ 11.5 VR units; see Methods) on 84% of trials. We performed spectral analyses of the iEEG signals during movement phases of the task for all hippocampal recording sites and used the MODAL oscillation-detection procedure^13^ to identify narrowband oscillations (see Methods). Overall, we observed hippocampal narrowband oscillations at frequencies in the range of 2–14 Hz (Fig. 2a), consistent with earlier findings^3,5,14–16^, with oscillations being most prevalent at ~3 Hz and ~8 Hz. For convenience, we label the frequencies of the oscillations in our data set as the low-theta (2–4 Hz) and high-theta (4–14 Hz) bands, although we acknowledge that some other studies have used the terms delta and alpha to refer to parts of these bands.

### Anatomical organization of hippocampal high and low theta

We next examined how the characteristics of the oscillations we identified varied with the location of the recording electrode along the hippocampal A–P axis. Many previous studies of hippocampal oscillations in rodents have focused on signals in the dorsal region, which is analogous to the posterior hippocampus in humans^11^, or on signals that are consistent across the length of the hippocampus^17^. However, a different line of work in humans^18–20^ and animals^21–23^ emphasized that there are substantial variations in function for neural activity recorded at different positions along the length of the hippocampus. This suggested to us that human hippocampal oscillations at different A–P positions could have distinct spectral and functional properties.

To examine the link between oscillation properties and intrahippocampal location in humans, we measured the A–P location of each hippocampal electrode in a subject-specific manner. We labeled the location of each electrode by measuring its relative position between the anterior and posterior extent of that subject’s hippocampus (see Methods). In this scheme, positions 0% and 100% correspond to electrodes at the anterior and posterior tips of the hippocampus, respectively. As seen in Fig. 2b, c, within individual subjects, we observed narrowband oscillations at various frequencies. Individual electrodes displayed oscillations at either one or two distinct frequency ranges during the task—we refer to these electrodes as single oscillators and dual oscillators, respectively (for example traces see Supplementary Fig. 1).

**Fig. 3:**
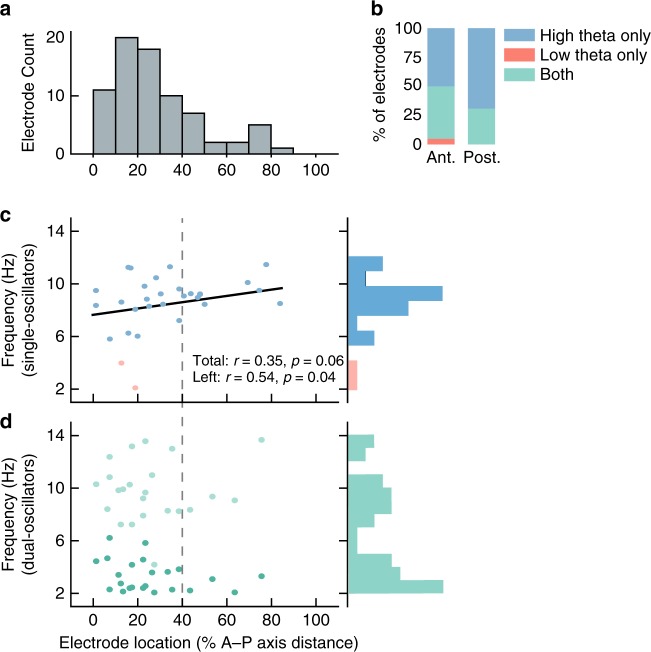
Oscillation properties across frequency and space. **a** Distribution of electrode locations along the hippocampus anterior–posterior (AP) axis. **b** Proportions of dual oscillators and single oscillators for anterior and posterior hippocampus. Source data are provided as a Source Data file. **c** Frequencies and hippocampal localizations of single oscillators across subjects. Black line indicates the fit between frequency and AP position for all high-theta electrodes in both hippocampi. Pearson *r* correlations between electrode location and frequency are reported for both hippocampi and for the left hippocampus alone. Gray dotted line indicates the split between anterior and posterior hippocampus. **d** Frequency and localization of dual oscillators. Dark green represents the slower oscillation.

Inspecting our data, we observed in many individuals that the frequency of the oscillations at a given hippocampal electrode correlated with its A–P location. These patterns resembled the A–P frequency gradients found from electrophysiological recordings from other brain areas^24–27^. Electrodes at posterior sites often showed oscillations at ~8 Hz. More anterior sites appeared to have oscillations at lower frequencies and more often showed two distinct oscillations (Fig. 3c, d).

We verified these observations quantitatively by analyzing oscillation mean frequencies across our complete data set. Although individual subjects generally were implanted with only a small number of hippocampal contacts, in aggregate our data set sampled 80% of the A–P length of the hippocampus (Fig. 3a). Every hippocampal electrode showed at least one narrowband oscillation within 2–14 Hz (Fig. 3b). 57% (30 of 53) of electrodes were single oscillators, which usually (93%) showed an oscillation in the high-theta (4–14 Hz) band (Fig. 3c). The remaining 43% (23 of 53) of electrodes were dual oscillators (Fig. 3d). In the posterior hippocampus, 69% of electrodes were single oscillators; whereas in the anterior hippocampus, 50% of electrodes were single oscillators and 50% were dual oscillators (Fig. 3b).

These patterns suggested to us that there could be a systematic relationship between the A–P position of a hippocampal recording electrode and the characteristics of the oscillations it recorded. Indeed, we found that A–P position alone was sufficient to significantly predict whether an electrode was a single or dual oscillator, with single oscillators being more prevalent in posterior locations (logistic regression, *p* = 0.02; Fig. 3b). Among the high-theta single oscillators, there was a trend for a gradient between oscillation frequency and A–P position, such that the specific high-theta frequency of an oscillation was greater for electrodes at more posterior locations (*r* = 0.35, *p* = 0.06; Fig. 3c). This frequency gradient was more clear in the left hippocampus than the right (left: *r* = 0.54, *p* = 0.04; right: *r* = 0.14, *p* = 0.19). Dual oscillators did not show a significant correlation between frequency and location for either the low- or high-theta bands (|*r*| < 0.2, *p*′s > 0.25; Fig. 3d). Together, these results indicate that human hippocampal theta is not a single unitary phenomenon, but that it instead shows a gradient in terms of its properties, with more posterior regions often showing a rodent-like theta, by exhibiting a single oscillation at a higher frequency. In contrast, hippocampal theta oscillations from more anterior locations were more likely to manifest either as two oscillations or as a single slower oscillation.

Building off our earlier work showing the functional lateralization of human theta oscillations^28^, here we went further to examine the spectral and anatomical properties of theta oscillations related to movement. We found that both left and right hemispheres displayed low- and high-theta oscillations (29 left electrodes, mean = 6.4 ± 0.5 Hz (mean ± SEM); 24 right electrodes, mean = 7.1 ± 0.5 Hz). Among the high-theta single oscillators, mean frequencies were significantly faster on the right hemisphere than the left (17 left electrodes; mean = 6.23 Hz, 13 right electrodes; mean = 8.12 Hz; *t*_34_ = 2.4, *p* = 0.02, unpaired *t* test). The high-theta oscillations on dual oscillators did not differ in frequency between the two hemispheres (*t*_27_ = 0.45, *p* = 0.65, unpaired *t* test).

We wished to confirm that our results were not biased by unbalanced electrode positioning across the hippocampus, either between hemispheres or across the dorsal–ventral axis. To analyze this, we compared the distributions of electrode locations across left vs. right hemispheres and across hippocampal subregions (see Supplementary Notes). There was not a significant difference in the distribution of electrode positioning between the left vs. right hemispheres (two-sample rank-sum test, *p* = 0.2), and we did not find significant frequency variations across subregions (one-way ANOVA; single oscillators: *F*_40_ = 1.8, *p* = 0.18; dual oscillators, *F*_47_ = 0.24, *p* = 0.79). Taken together, these results indicate that our findings of variations in the properties of hippocampal oscillations along the A–P axis is not an artifact of a difference in electrode positioning.

To better understand the phenomenon of dual-oscillator electrodes, we examined the relationship between their lower- and higher-theta oscillations. We first considered the possibility that there was a link between the particular frequencies of the oscillations that appeared at individual dual oscillators. This could have been the case, for example, if one electrode that showed two apparent oscillations was actually recording an oscillation with a non-sinusoidal waveform^29^, and this caused the power spectrum to show the oscillation as well as its harmonic. However, there was no correlation between the frequencies of the high and low oscillations at individual dual oscillators (*p* = 0.85, permutation test), indicating that the faster oscillations at these sites were not harmonics of the slower ones. In addition, we compared the timing of the occurrence of the different oscillations on dual oscillators and found a tendency for these electrodes to measure oscillations at both bands simultaneously (Wilcoxon signed-rank test, *p* = 0.03; see Supplementary Fig. 2A). Together, these results indicate that signals at dual oscillators reflect distinct oscillations with a moderate tendency to co-occur in time. Finally, some subjects had multiple distantly spaced electrodes along the A–P axis. This enabled us to test whether oscillations measured at different A–P locations were temporally related (i.e., through volume conduction). We conducted an analysis of theta-phase synchrony separately for the simultaneously recorded electrode pairs that exhibited low- and high-theta oscillations, and found that volume conduction did not explain our findings (see Supplementary Notes; Supplementary Fig. 3). Instead, we detected a pattern of characteristic phase lags that indicated that the theta oscillations were traveling waves that tended to propagate from sites with faster to slower oscillation frequencies, consistent with coupled-oscillator models^26,30,31^.

### Analysis of theta-bout duration

**Fig. 4:**
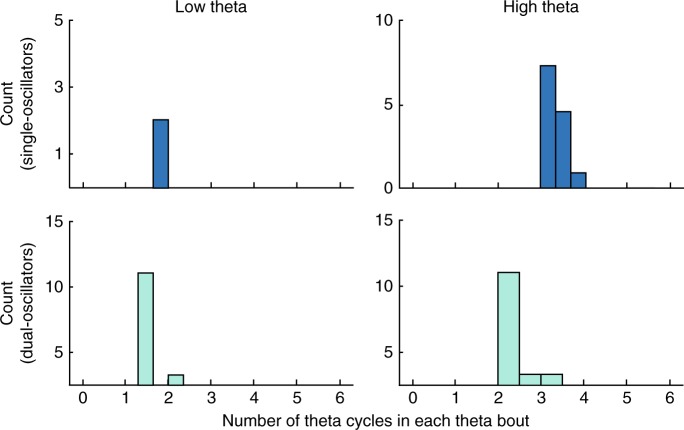
Number of theta cycles within individual theta oscillation bouts. Histograms showing the distributions of mean cycle lengths of the bouts of theta oscillations from individual electrodes. Individual plots show these distributions separately for low- and high-theta oscillations from single- and dual-oscillator electrodes.

Earlier studies showed that theta oscillations in both humans and monkeys appeared in transient bouts^14,32,33^. These human theta bouts were shorter in duration compared with rodent theta oscillations, which often persisted for many seconds^1^. To compare our results with signals in rodents, we measured the mean duration of continuous oscillatory cycles of theta signals from individual electrodes in the low- and high-theta bands, for single- and dual-oscillator electrodes (Fig. 4). Here, prior to statistical analysis, we first averaged oscillatory signals that were simultaneously recorded from nearby electrodes in a subject (see Methods).

Individual subjects showed a range of mean theta-bout durations. The mean bout duration was longer for high- than low-theta oscillations (2.73 vs. 1.37 cycles, respectively; *t*_38_ = 12.0, *p* < 10^−13^). Within the high-theta band, we observed longer theta bouts at single than dual-oscillator electrodes (3.0 vs. 2.43 cycles, respectively; *t*_25_ = 6.0, *p* < 10^−5^). The relatively long duration of high-theta in single oscillators makes it the closest human analog to hippocampal theta in moving rodents; however, it is still dramatically briefer than rodent theta, which can have bouts that last many seconds^1,32,34^.

### High-theta frequency correlates with movement speed

In rodents, the instantaneous frequency of the hippocampal theta oscillation correlates with the speed of running^34^. Further, in both humans and rodents theta power correlates with speed^5,23,34^. These results have been interpreted to indicate that theta oscillations have a general role related to multiple aspects of spatial cognition^14,35,36^.

Building off this work, we tested for correlations between movement speed and theta frequency to specifically distinguish the functional role for human hippocampal oscillations in spatial processing. To do this, at each electrode we measured the precise frequency of the oscillations in each of the three movement epochs per trial, in which subjects were moved at a particular fixed speed along the virtual track. Next, we subsampled the data to randomly select only one movement epoch per trial (see Methods). Finally, for each electrode, we computed the correlation across epochs between the movement speed and the oscillation frequency.

**Fig. 5:**
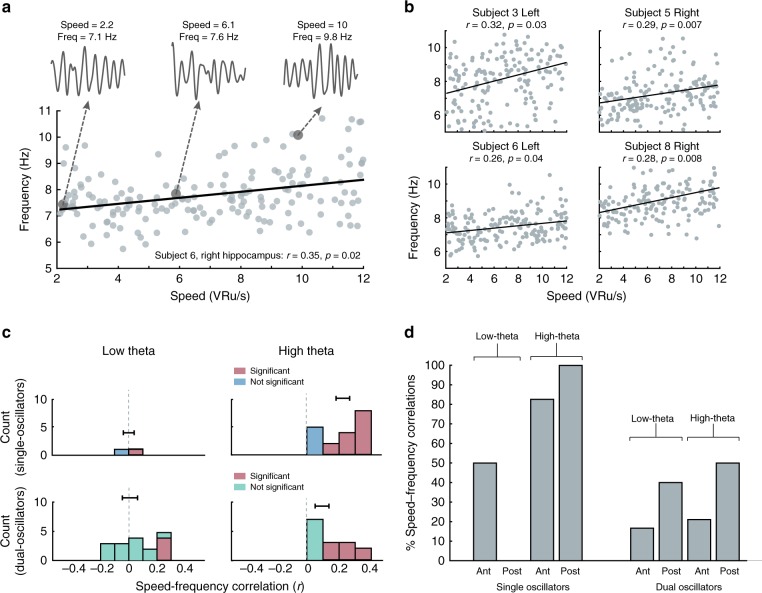
Analyses of the relation between theta frequency and movement speed. **a** An example electrode with a positive high-theta frequency–speed correlation. Two-second trace of filtered hippocampal oscillations during slow, medium, and fast speeds. Pearson *r* correlation is reported between speed and frequency, *r* = 0.35, *p* = 0.02. **b** Example electrodes from both left and right hippocampus that display significantly positive high-theta speed–frequency correlations. Pearson *r* correlations are reported between speed and frequency for each electrode. **c** Histogram of correlation coefficients for single and dual oscillators, separately aggregated for low- and high-theta bands. Significant correlations indicated in red. Error bars are SEM. Low-theta single oscillators: *n* = 2 electrodes. Low-/high-theta dual oscillators: *n* = 19 electrodes. High-theta single oscillators: *n* = 19 electrodes. **d** Percentage of electrodes in each hippocampal region with a significant positive correlation between movement speed and frequency for both low- and high-theta bands. Data in panels **c** and **d** were computed after subsampling to include only one movement epoch per trial. Source data are provided as a Source Data file.

Many electrodes with high-theta oscillations showed positive correlations between frequency and movement speed. Figure 5a, b illustrates this pattern of results for five example electrodes. We found that the mean correlation between movement speed and oscillation frequency was significantly positive for high-theta oscillations (Fig. 5c, right), both when this signal was observed on single as well as dual oscillator electrodes (both *p*′s < 0.006). The mean speed–frequency correlation was significantly larger for single than dual high-theta oscillators (*t*_36_ = 2.42, *p* = 0.02). This effect was also statistically significant on the single-electrode level. Of the 19 high-theta single oscillators, 13 (68%) showed a significant (*p* < 0.05) speed–frequency correlation, which was more than expected by chance (*p* < 10^−5^, binomial test). Similarly, of 19 dual oscillators, 7 (37%) showed a significant high-theta speed–frequency correlation (*p* < 10^−5^, binomial test).

In contrast to the high-theta band where we found robust positive speed–frequency correlations, the low-theta band showed a different pattern, where speed–frequency correlations were not significantly greater than zero (*p*′s > 0.05; Fig. 5c, left). Similarly, at the single-electrode level, of the 20 electrodes with low-theta oscillations (including both single and dual oscillators), only 5 (25%) showed significant speed–frequency correlations, which is significantly less than the proportion of electrodes with high-theta oscillations that showed this effect (*z*-test, *z* = 2.4, *p* = 0.008).

Finally, given the multiple factors that correlated with theta’s properties—movement speed, electrode location, and oscillation frequency—we performed a multivariate analysis to disentangle the interrelations between these factors. We used a two-way ANOVA to establish how the presence of speed–frequency correlations, averaged within subject, varied across region (anterior/posterior), band (high/low theta), and their interaction (Fig. 5d). For single oscillators, we found a significant interaction effect (*p* = 0.03), but no significant main effects (A–P region: *p* = 0.09, frequency: *p* = 0.37), thus confirming our interpretation that the prevalence of speed–frequency effects is significantly greater for posteriorly located high-theta single-oscillator electrodes (Fig. 5d). We performed a similar analysis for dual oscillators, and found no significant main or interaction effects (all *p*′s > 0.05). This result indicates that high-theta oscillations at single oscillators in the posterior hippocampus are more closely tied to spatial processing. More broadly, this supports the view that high-theta oscillations in the posterior hippocampus are related to the theta oscillations that are commonly seen in rats during movement.

## Discussion

By recording along the length of the hippocampus from subjects performing a virtual-reality spatial task, we identified multiple patterns of hippocampal theta-band oscillations with separate functional and anatomic properties. These findings suggest that human hippocampal theta oscillations are more than just a slower and noisier analog of the single theta oscillation seen in rodents, but that they instead exist in multiple forms. Specifically, we identified high (~8 Hz) theta oscillations in the posterior hippocampus that varied their frequency with the speed of movement during virtual navigation, similar to the theta oscillations seen in rodents^34^. We also found that humans have slower (~3 Hz) hippocampal theta oscillations with distinct functional and anatomical properties. In conjunction with earlier work linking human low theta to memory^15,28,37^, our results suggest that high- and low-theta oscillations represent distinct functional network states. In this way, our work supports the broader view that the brain can exhibit distinct oscillatory states related to different behaviors^38^. Further, because the prevalence of spatially relevant single-oscillator electrodes differed along the A–P length of the hippocampus, our findings provide electrophysiological evidence for a functional gradient across the hippocampus. This is a substantial difference compared to rodents, which usually are described as showing a single theta rhythm across the hippocampus (e.g., refs. ^17,39^; but see ref. ^40^).

Previous work on human hippocampal oscillations generally emphasized that rhythms at ~1–5 Hz were more common (for review, see ref. ^6^). Our study has several distinctive methodological features that could explain why we observed a different pattern compared to the earlier literature, including a greater prevalence of hippocampal oscillations at faster frequencies. Although not all studies report the intrahippocampal locations of recording electrodes, it seems that they usually most extensively sampled anterior areas of the hippocampus^5,32^. By contrast, we measured each electrode’s A–P location and included greater electrode coverage in middle and posterior sections of the hippocampus, which were the regions that more specifically showed single high-theta oscillations. This increased posterior hippocampal coverage is likely the result of the use of stereotactic electroencephalographic (sEEG) recording electrodes, which have recently become more commonly used for clinical epilepsy mapping^19^.

An additional differentiating feature of our study was the design of our behavioral task. Rather than allowing the subject to control their own movement with a fixed top speed as in earlier studies^3,28,41^, here the task automatically changed the subject’s speed randomly, at random times. Given this unpredictable movement, to perform the task well, subjects could not predict their location based on timing and had to continually attend to their view of the spatial environment. We hypothesize that this increased spatial attention increased the prevalence of neural oscillations related to spatial processing. Accordingly, the relatively large prevalence of high-theta oscillations that we observed is consistent with the idea that this signal is particularly important for spatial processing and thus functionally analogous to the Type 1 theta observed in rodents during movement^42–46^.

Having demonstrated that human slow and fast theta oscillations differ functionally and anatomically, our results raise the question of the functional role of human low theta. One potential explanation is that this signal, which we more often found in the anterior hippocampus, is related to the Type 2 theta oscillations that had been characterized previously in rodents. Consistent with this interpretation, recent work showed that slower, Type 2 theta oscillations in rodents can be generated by a distinct network of interneurons in the ventral hippocampus^47^. Type 2 theta oscillations appear most strongly at ~4 Hz when rodents are stationary and are traditionally associated with anxiety (e.g., ^48,49^; but see ref. ^50^). In contrast, newer data from humans link oscillations in this low-theta band to memory processing^15,19,28^. Therefore, we suggest that the low-theta oscillations we observed are indeed related to the rodent Type 2 theta, but that this signal in humans has a broader functional role extending beyond anxiety, perhaps including episodic memory and other types of cognitive processes that more specifically involve the anterior hippocampus^51,52^.

A notable feature of our findings is identifying many dual-oscillator electrodes, which seem to reflect hippocampal networks that are capable of exhibiting both low- and high-theta signals. The existence of these dual oscillators may be important theoretically because the hippocampus in both rodents and humans is known to exhibit theta-traveling waves that propagate in a posterior-to-anterior (in humans) or dorsal-to-ventral (rodent) direction^17,31,53^. One potential mechanism for hippocampal traveling waves is a network of weakly coupled oscillators^26,30^. The multiple oscillations shown by dual oscillators may reflect the underlying independent oscillators that can lead to the generation of traveling waves when the phase coupling between them is increased. In particular, by showing that oscillatory phase progresses from locations with fast oscillators to those with slower ones (Supplementary Fig. 3), our results are consistent with the predictions of this model^30^ and earlier results^26,31,54^.

A key result from our work is showing that high-theta oscillations appear in the human hippocampus during movement in virtual reality. Two recent studies measured human hippocampal oscillations from people walking in the physical world and reported high-theta oscillations (e.g., ^7,8^; but see ref. ^55^). These results were interpreted to suggest that virtual navigation relies on a fundamentally different, lower-frequency oscillatory network state compared with real-world navigation^9^. However, it should be noted that at least one of the studies that previously showed high-theta oscillations in real-world navigation showed examples of these patterns at relatively posterior locations^8^. By demonstrating that humans can show high theta during virtual reality, our results suggest a different view. We propose that theta oscillations at various frequencies can be prevalent in both virtual and real spatial environments, with the dominant oscillatory frequency being closer to the high-theta range during moments of relatively high spatial demands. It is possible that the earlier VR studies showed relatively less high-theta because their associated tasks required less spatial attention. Overall, both our current findings and this earlier literature lend support to the notion that the human anterior and posterior hippocampi, respectively, are implicated in low- and high-theta oscillations that each have different behavioral properties^11,22,56^.

One reason why theta oscillations are thought to be important functionally is that they coordinate large-scale brain-wide networks, including synchronizing cortical–hippocampal interactions^57^. Therefore, given that we showed that the human hippocampus exhibits two separate theta oscillations in a single task, an important area of future work will be to understand the potential relation of each of these signals to brain-wide neocortical dynamics^58,59^. In particular, it is notable that the posterior-to-anterior fast-to-slow oscillatory gradient that we observed in the hippocampus seems to match the direction of the frequency gradient that has also been found in the neocortex^25–27,52,60^. In light of this frequency-wise correspondence between the hippocampal and neocortical oscillations, we hypothesize that there could also be a functional relation between these oscillations. Specifically, one possibility is that there could be a functional link between so-called alpha rhythms in the posterior neocortex and high-theta oscillations in the posterior hippocampus. Supporting this idea, it is notable that we found a correlation between high-theta frequency and movement speed, because it suggests that this oscillation could relate to the kinds of visuospatial processes that are commonly associated with occipital alpha oscillations^61–65^. Consistent with this view, recent work on cortical idling demonstrated a potential link between hippocampal and occipital oscillations by showing that 8–12-Hz power in both regions increased with eye closure^66^. Furthermore, new work has identified molecular similarities between the anterior hippocampus and frontal cortical regions, and between the posterior hippocampus and occipital cortical regions^56^. In light of these convergences, it seems promising for future studies to probe links between hippocampal high-theta and visual alpha oscillations.

More broadly, our finding that different theta frequencies are preferentially associated with anterior and posterior processes is notable given the predominant involvement of the frontal and occipital lobes in high-level and sensory processing, respectively. Along with the different functional correlations we found for low- and high-theta frequencies, these results are consistent with the idea that oscillations at varying frequencies reflect distinct hippocampal–neocortical interactions related to different functions^38,59^. This multiplicity of human theta patterns—across high and low frequencies—could be a critical component in allowing the human hippocampus to coordinate a diverse set of brain-wide neural assemblies to support various types of behaviors, including spatial navigation, memory, and other cognitive processes.

## Methods

### Subjects

Fourteen subjects (eight males and six females, age range 23–49) at four hospitals (Thomas Jefferson University Hospital, Columbia University Medical Center, University of Texas Southwestern Medical Center, and Emory University Hospital) undergoing treatment for medication-resistant epilepsy participated in our study. Neurosurgeons implanted these subjects with clinical depth electrodes for functional mapping and the localization of seizure foci. Implantation sites were determined solely by clinical teams, though electrodes were often placed in medial temporal lobe regions that are of interest experimentally. Research protocols were approved by the institutional review boards at each participating hospital, and informed consent was obtained from all subjects. Previous work utilizing scalp EEG recordings^67^ has reported that theta oscillatory activity varied with age and sex. However, here we did not find a significant relation between theta frequency and age (*r* = −0.09, *p* = 0.90) or sex (*t*_11_ = 0.97, *p* = 0.35, unpaired *t* test). This difference suggests that the hippocampal oscillations that are the focus of our study differ from the neocortical signals measured with scalp EEG.

### Task

The subjects in our study performed a new spatial memory task, which we specifically designed to encourage subjects to pay attention to their location in the virtual environment by varying their movement speed randomly. This distinctive design prevented subjects from utilizing a timing-based strategy to perform the task, such as by remembering each object’s latency since the beginning of movement. We hypothesized that this task design had the potential to elicit more reliable hippocampal activity related to spatial processing than previous studies of human navigation^12^. Because the subjects in our study were undergoing continuous monitoring for epileptiform activity, we were limited to studying virtual navigation, as subjects remained in their hospital bed throughout testing.

In the task, subjects were moved along the length of a virtual-reality (VR) track, which we defined as having a length of 70 virtual-reality units. The ground was textured to mimic asphalt, and the track was surrounded by stone walls (see Fig. 1). On each trial, subjects were placed at the beginning of the track, and they began each trial by pressing a button on a game controller. Next, a four-second-long countdown timer appeared. After the countdown, subjects were moved forward along the track. Within each third of the track, subjects were moved at a constant speed, which was chosen randomly from a uniform distribution between 2 and 12 VR units/second. Locations where speed changes began are indicated by the light gray shading in the schematic shown in Fig. 1c. When speed changes occurred, acceleration occurred gradually over the course of one second to avoid jarring transitions.

During movement, the subjects’ goal was to mark the location of a hidden object. The first two times that the subject traveled down the track, the object’s location was visible (Fig. 1a). On subsequent trials, the object was invisible, and subjects were instructed to press the button on the controller when they believed they were at the correct location (Fig. 1b). The closer the subject pressed the button to the correct location, the more points they received (as indicated in the top right of the display), thus encouraging careful attention to current location in the environment. Subjects were also required to press the button when they approached the end of the track where the ground was colored red to ensure that they were attentive during the trial. Possible object locations are indicated by the dark gray shading in Fig. 1c.

Each trial consisted of the subject traveling a single time down the track, either encoding or retrieving object location. Within each trial, the task would automatically change the subject’s speed at each of two possible speed-change regions (Fig. 1c), such that the subject’s path down the track consisted of three constant speed regions. The focus of this study was to analyze human hippocampal correlates of movement, rather than memory or task performance. Thus, we analyzed all time points while subjects were in motion, regardless of performance, including both encoding and retrieval trials. We classified correct trials as trials where subjects had a response–object error distance of less than 11.5 VR units. This value represents the average error that subjects would display if they responded at the midpoint of the track each time^12^.

### Electrophysiology

We recorded subjects’ intracranial electroencephalographic (iEEG) data from implanted depth electrodes via the clinical or research recording systems present at the participating hospitals (Nihon Kohden; XLTEK; Neuralynx; Blackrock). Data were recorded at a sampling rate of either 1000 or 2000 Hz. iEEG signals were initially referenced to common intracranial or scalp contacts, and were subsequently re-referenced using an anatomically weighted referencing scheme prior to analysis. Data were notch filtered at 60 Hz using a zero-phase-distortion Butterworth filter to remove line noise prior to subsequent analyses. iEEG recordings were aligned to the behavioral task laptop via synchronization pulses sent to the recording system.

### Electrode localization

Our data analyses were designed to test how the functional and electrophysiological properties of human theta oscillations varied along the hippocampal A–P axis. To study electrode’s anatomical features, we localized depth electrodes for each subject using an established semi-automated image processing pipeline^68^. To delineate the hippocampus, we applied the Automatic Segmentation of Hippocampal Subfields multi atlas segmentation method to pre-implantation high-resolution hippocampal coronal 3T T2-weighted and whole-brain 3D T1-weighted scans. Electrode contact coordinates derived from post-implantation CT scans were then co-registered to the segmented MRI scans using Advanced Normalization Tools^69^, and anatomic locations were automatically generated. A neuroradiologist reviewed and confirmed contact locations based on the co-registered source images. Electrodes were assigned normalized locations along the hippocampal axis by determining the coronal slice containing the center of the contact and measuring relative to the first and last MRI slice containing the hippocampus. For specific subjects, a neuroradiologist generated transparent 3D surface renderings of the subject's hippocampal segmentation and the corresponding co-registered electrode contacts. Here, we only analyzed electrodes located within the hippocampal formation (CA1, CA2, subiculum, and dentate gyrus). For the majority of our analyses, we analyzed electrode location as a continuous variable along the hippocampal A–P axis; however, when it was more convenient to refer to anterior and posterior labeling, we utilized 40% as the division point, based on the midpoint of our coverage, to allow adequate statistical power for data analyses. If two or more neighboring electrodes in one subject were located in nearby slices (<10% of the hippocampal A–P axis distance away from each other), and exhibited a similar oscillation frequency (within 2 Hz) during movement, all but one of these electrodes were dropped for all analyses.

### Spectral analysis

Due to the variability of human neuronal oscillations^15,31^, our analyses examined the spectral features of the oscillations at each electrode at a high resolution to identify frequency bands that are customized for each subject and electrode^26,70^. This approach differs from the one used in our earlier work, which utilized fixed frequency bands across subjects. To achieve this high-frequency resolution, we followed the MODAL algorithm^13^. The first step of this algorithm is to exclude epochs of the data that could potentially result from epileptic activity^71^. Then, the algorithm defines relevant frequency bands as those frequencies where the measured oscillatory power exceeds one standard deviation above the background 1/*f* spectrum. This criterion ensures that our results were not driven by spurious background noise information. MODAL then computes the instantaneous frequency and phase for each frequency band, but only when the local power spectrum (computed in 10 s, nonoverlapping windows) indicated a local power peak at that band.

When examining frequency band characteristics across the data, we noticed that every electrode exhibited either one or two distinct oscillations at frequency bands between 2 and 14 Hz. We called electrodes that only exhibited a single oscillation throughout the task single oscillators while we called those that exhibited two oscillations dual oscillators. For an electrode to be designated as a dual oscillator, the edges of the two frequency bands detected by MODAL had to differ by at least 0.5 Hz. For analyses where we specifically report low-theta and high-theta results across electrodes, we classified low-theta oscillations as those <4 Hz, and high-theta oscillations as those >=4 Hz. We defined an oscillatory bout as sequences of consecutive millisecond time points of any length where at least one oscillation was present.

We performed a series of analyses comparing how these detected oscillations related to features of the subject’s movement. Each trial within the task consisted of three intervals that each had a constant speed of movement (Fig. 1). We computed the particular oscillation frequency for each movement interval by first using MODAL to measure the instantaneous frequency of the iEEG signal at each timepoint throughout the interval. Then, we computed a histogram of the distribution of frequencies (0.1-Hz bins), identified the single most often occurring frequency (i.e., the mode), and used this value to summarize the oscillatory activity in that interval. For our analysis of speed–frequency correlations (Fig. 5a–c), we randomly chose only a single-speed period from each trial to analyze to ensure that all speed–frequency correlations arose from independent observations. For the analyses of oscillatory bouts (Fig. 4) and frequency–speed correlations (Fig. 5), we wished to ensure that our results were not influenced by subjects who had multiple electrodes at similar A–P locations. Therefore, we performed a group-level analysis where each subject contributed only a single mean value per frequency/region, for each of the low-anterior, low-posterior, high-anterior, and high-posterior categories. All data analysis was completed in MATLAB 2017b.

### Reporting summary

Further information on research design is available in the Nature Research Reporting Summary linked to this article.

## Supplementary information

Supplementary Information

Reporting Summary

## Data Availability

The data sets generated during and analyzed during this study are available from the corresponding author on reasonable request. The source data underlying Figs. 3b and 5d are provided as a Source Data file.

## Source Data

Source Data

## Code availability

The code used to analyze data during the current study are available from the corresponding author on reasonable request.
