# Supplementary material for: Functionally distinct high and low theta oscillations in the human hippocampus
Source: Nat Commun. 2020 May 18;11:2469. doi: 10.1038/s41467-020-15670-6 (PMC7235253; doi:10.1038/s41467-020-15670-6)
Supplement: Supplementary file 1 — Supplementary Information [file 41467_2020_15670_MOESM1_ESM.pdf]

Functionally distinct high and low theta oscillations in the human hippocampus

Goyal et al.

Supplementary Notes

Analysis of Hippocampal Subregions. We considered the possibility that our results could have been affected by other anatomical factors besides A–P location. To test for an effect of the electrode's dorsal–ventral (D–V) position, we compared the relation between the D-V location (calculated for each electrode in the same manner as A-P location - see *Methods*) of each electrode within the hippocampus and the frequency of the measured oscillations, both for single and dual oscillators. We did not find a significant relation between frequency and D-V location for low- or high-theta oscillations for either single or dual oscillators (all $p's > 0.05$). To further probe potential effects of electrode positioning, we performed a new analysis where we manually identified each electrode's subregion. Then, to aggregate data across subjects, we compared signals between these subregions (CA1, CA2, DG, and subiculum). This procedure allowed us to examine potential effects of D-V location while accounting for inter-individual morphological differences. For both single and dual oscillators, theta frequency did not significantly change across subregion (single oscillators: $F_{40} = 1.8$, $p = 0.18$; dual oscillators, $F_{47} = 0.24$, $p = 0.79$). Together, these results indicate that our primary results of theta frequency shifting with A–P position (Fig. 3) are not confounded by variations related to subregion or D–V location.

Timing analysis between dual oscillator bouts. We characterized the temporal relationship between the two oscillations that appeared on dual oscillator electrodes during movement to determine whether they tended to occur simultaneously or at alternate timepoints (Supplementary Figure 2). For this analysis, we identified all oscillations on each band for dual oscillators that were at least 2 cycles in length. Then, for each electrode, we labeled each timepoint with two binary variables, indicating the oscillation's presence or absence for each band. Finally, for each electrode, we computed the ϕ correlation coefficient between the binary variables for each of the two bands—a positive ϕ would indicate that an electrode tended to exhibit oscillations at both bands simultaneously. Comparing the distribution of ϕ values across electrodes, we found the mean ϕ was significantly positive (mean $\phi = 0.07$, Wilcoxon signed-rank test, $p = 0.03$; Supplementary Figure 2A). This result indicates that dual oscillators tended to show oscillations at both of its bands simultaneously.

Low- and High-Theta Phase Analysis. We wanted to understand the spatial organization of theta hippocampal oscillations at different frequencies along the A–P axis, inspired by our earlier finding of theta traveling waves that propagate from regions with fast to slow relative frequencies [1]. To probe this issue, we identified data from subjects who had pairs of simultaneously implanted electrodes spanning at least 30% of their hippocampus. We then analyzed the instantaneous phase at each electrode and computed the mean phase difference between those electrodes in each trial. We then tested whether the theta phases from each electrode in the pair were correlated using circular statistics [2]. The results were as follows: 21% of high-theta electrode pairs showed theta oscillations with independent phase patterns (Rayleigh test for phase uniformity, $p > 0.05$). 45% of pairs showed high-theta oscillations that were synchronized (Rayleigh test $p < 0.05$) with a $0° \pm 10°$ phase shift—this indicates that synchronized theta oscillations appeared in both electrodes with the same timing, as predicted by volume conduction. 33% of electrode pairs showed high-theta oscillations with a consistent phase shift between electrodes, which is suggestive of a traveling wave.

We examined the propagation patterns from the electrode pairs with consistent phase shifts to test a prediction of the weakly-coupled-oscillator model [3], that traveling waves would propagate towards regions with slower oscillation frequency [1]. We tested for this pattern in our data by measuring the sign of the phase shift across the electrode pair and testing whether the sign of this phase shift

correlated with the electrodes' difference in relative frequency. Consistent with the coupled oscillator model, we found that the direction of traveling wave propagation followed the orientation of local frequency gradients. For the pairs where the more posterior electrode showed a faster oscillation frequency, 65% of the time, the pair showed traveling waves propagating in a posterior-to-anterior direction (Supplementary Figure 3B–C). This directionality was less consistent for the electrode pairs that had a frequency gradient with the reverse orientation ($\chi^2 = 3.4$, $p = 0.06$), indicating that having posterior-to-anterior traveling theta waves is correlated with having faster oscillations towards the posterior hippocampus.

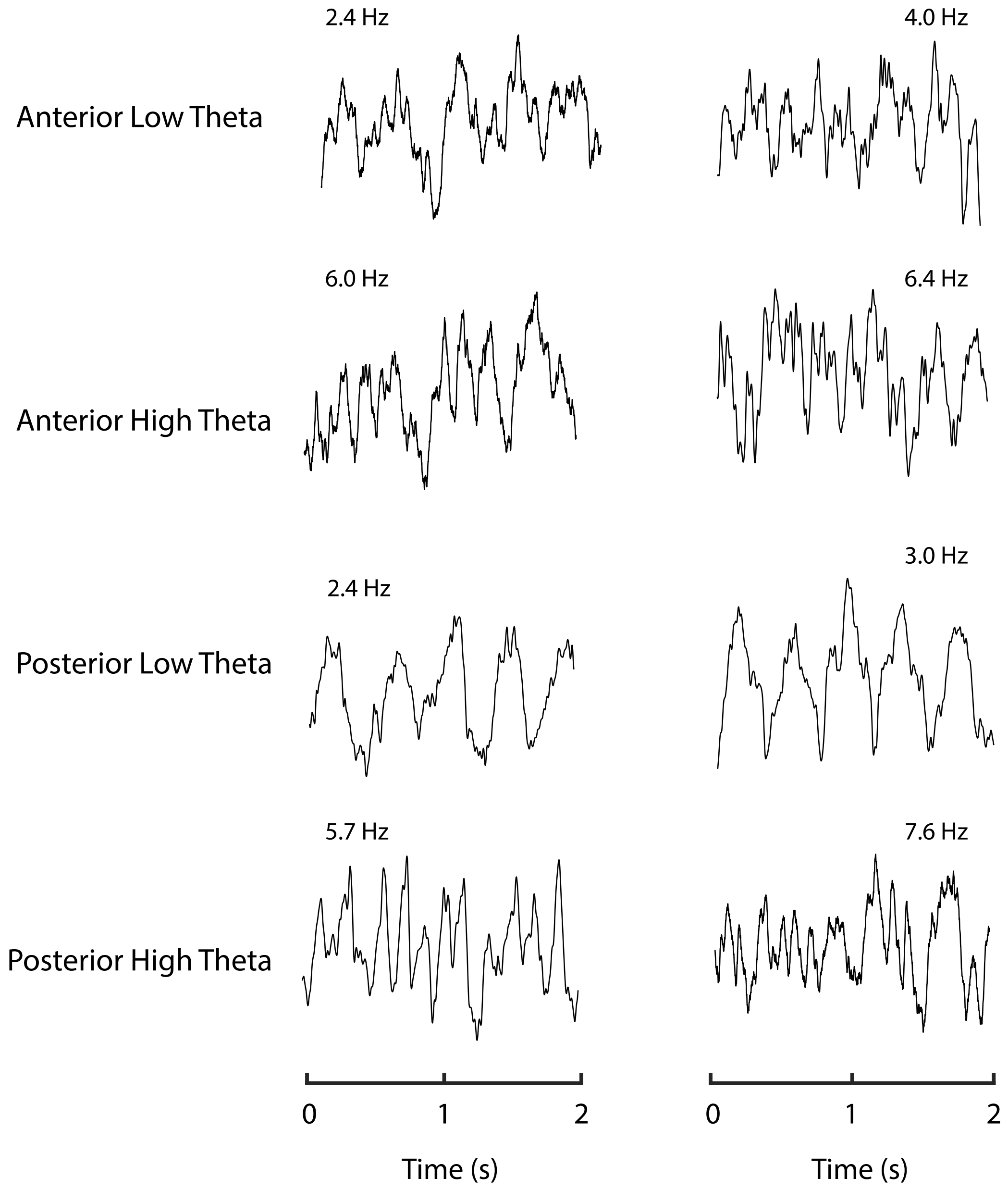

Supplementary Figure 1: Raw EEG Traces Raw EEG traces from different subjects within our dataset. Data were lowpass filtered at 55 Hz to filter out line-noise.

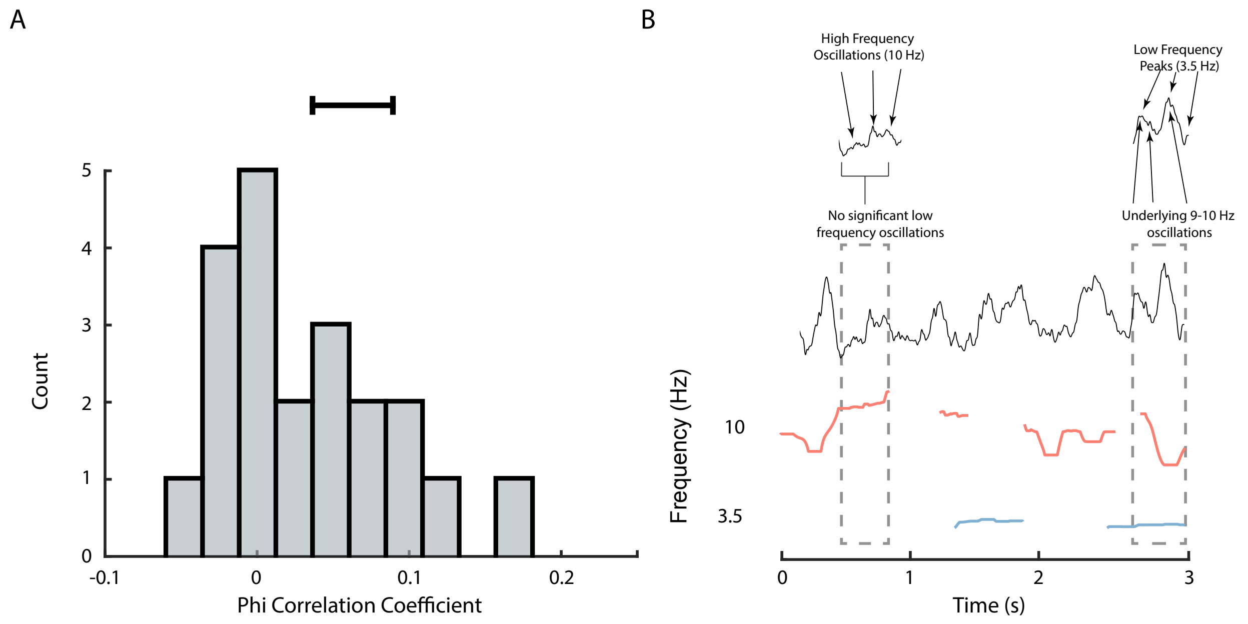

Supplementary Figure 2: Dual oscillator bout correlation A. Histogram of ϕ correlation coefficients from all dual oscillators within our dataset. The distribution is significantly greater than 0 (Two-sided Wilcoxon signed-rank test, $p = 0.03$). Confidence interval of mean of distribution computed via SEM. **B.** Raw EEG traces above high-theta and low-theta prevalences in a continuous signal of 3 seconds from an electrode within our dataset that exhibits characteristic periods where the low- and high-theta bouts appear separately (left) and co-occur (right).

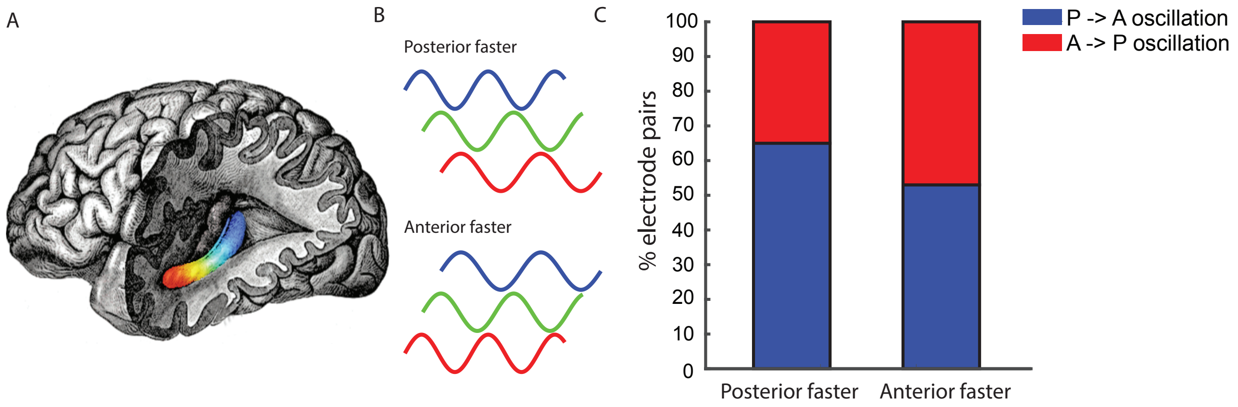

Supplementary Figure 3: Phase analysis A. Schematic of the human hippocampus, with colors signifying location along the Anterior–Posterior axis (red is anterior, blue is posterior). **B.** Example iEEG traces from electrodes within a single subject at similar frequencies demonstrating phase shifts (not real data). **C.** For all electrode pairs that exhibit consistent phase shifts, the plot indicates the percent of electrode pairs that exhibit anterior-to-posterior traveling waves (red) and posterior-to-anterior traveling waves (blue), with electrode pairs separated by the site that showed the faster oscillation.

Supplementary References

[1] Honghui Zhang, Andrew J Watrous, Ansh Patel, and Joshua Jacobs. Theta and alpha oscillations are traveling waves in the human neocortex. *Neuron*, 98(6):1269 – 1281.e4, 2018.

[2] P. Berens. Circstat: A matlab toolbox for circular statistics. *Journal of Statistical Software*, 31(10), 2009.

[3] G.B. Ermentrout and D. Kleinfeld. Traveling Electrical Waves in Cortex Insights from Phase Dynamics and Speculation on a Computational Role. *Neuron*, 29(1):33–44, 2001.
