# Supplementary material for: Functionally distinct high and low theta oscillations in the human hippocampus
Source: Nat Commun. 2020 May 18;11:2469. doi: 10.1038/s41467-020-15670-6 (PMC7235253; doi:10.1038/s41467-020-15670-6)
Supplement: Supplementary file 2 — Reporting Summary [file 41467_2020_15670_MOESM2_ESM.pdf]

nature research

Corresponding author(s): Joshua Jacobs

Last updated by author(s): 03-16-2020

Reporting Summary

Nature Research wishes to improve the reproducibility of the work that we publish. This form provides structure for consistency and transparency in reporting. For further information on Nature Research policies, see Authors & Referees and the Editorial Policy Checklist .

Statistics

For all statistical analyses, confirm that the following items are present in the figure legend, table legend, main text, or Methods section.

n/a	Confirmed	
☐	☒	The exact sample size (*n*) for each experimental group/condition, given as a discrete number and unit of measurement
☐	☒	A statement on whether measurements were taken from distinct samples or whether the same sample was measured repeatedly
☐	☒	The statistical test(s) used AND whether they are one- or two-sided<br>*Only common tests should be described solely by name; describe more complex techniques in the Methods section.*
☒	☐	A description of all covariates tested
☐	☒	A description of any assumptions or corrections, such as tests of normality and adjustment for multiple comparisons
☐	☒	A full description of the statistical parameters including central tendency (e.g. means) or other basic estimates (e.g. regression coefficient) AND variation (e.g. standard deviation) or associated estimates of uncertainty (e.g. confidence intervals)
☐	☒	For null hypothesis testing, the test statistic (e.g. *F*, *t*, *r*) with confidence intervals, effect sizes, degrees of freedom and *P* value noted<br>*Give P values as exact values whenever suitable.*
☒	☐	For Bayesian analysis, information on the choice of priors and Markov chain Monte Carlo settings
☒	☐	For hierarchical and complex designs, identification of the appropriate level for tests and full reporting of outcomes
☐	☒	Estimates of effect sizes (e.g. Cohen's *d*, Pearson's *r*), indicating how they were calculated

Our web collection on statistics for biologists contains articles on many of the points above.

Software and code

Policy information about availability of computer code

Data collection	iEEG data from subjects were obtained via the clinical or research recording system of the hospital at which the patient was being treated (Nihon Kohden; XLTEK; Neuralynx; Blackrock)
Data analysis	All data analysis was done in MATLAB 2017b.

For manuscripts utilizing custom algorithms or software that are central to the research but not yet described in published literature, software must be made available to editors/reviewers. We strongly encourage code deposition in a community repository (e.g. GitHub). See the Nature Research guidelines for submitting code & software for further information.

Data

Policy information about availability of data

All manuscripts must include a data availability statement. This statement should provide the following information, where applicable:

- Accession codes, unique identifiers, or web links for publicly available datasets
- A list of figures that have associated raw data
- A description of any restrictions on data availability

The datasets generated during and analyzed during the current study are available from the corresponding author on reasonable request. The source data underlying Figures 3B and 5D are provided as a Source Data file.

Field-specific reporting

Please select the one below that is the best fit for your research. If you are not sure, read the appropriate sections before making your selection.

Life sciences study design

All studies must disclose on these points even when the disclosure is negative.

Sample size	Our data consisted of 14 subjects, in whose hippocampi a total of 90 electrodes were implanted. No sample size calculations were performed. This sample size was sufficient to provide adequate statistical power to all analyses within our manuscript.
Data exclusions	If two or more neighboring electrodes in one subject were located in nearby slices (less than 10% of the hippocampal A-P axis distance away from each other), and exhibited a similar oscillation frequency (within 2 Hz) during movement, all but one of these electrodes were dropped for all analyses.
Replication	All findings reported in this study were obtained using deterministic algorithms, and will thus produce the same result each time they are run.
Randomization	We conducted our analyses in both electrode-wise and subject-wise manners. Electrodes were allocated into anterior and posterior hippocampi regions, to low- and high-theta oscillation bands, and to single and dual oscillator categories. In subject-wise analyses, each subject's electrodes were allocated into low-anterior, low-posterior, high-anterior, and high-posterior categories. Randomization is not relevant, as all subjects/electrodes were placed into groups based on their biologic characteristics, and were not allocated through some subjective or random means.
Blinding	Blinding was not relevant to our study, as the outcomes of the study will not directly impact the patients from whom the data was acquired.

Reporting for specific materials, systems and methods

We require information from authors about some types of materials, experimental systems and methods used in many studies. Here, indicate whether each material, system or method listed is relevant to your study. If you are not sure if a list item applies to your research, read the appropriate section before selecting a response.

Materials & experimental systems

n/a	Involved in the study	
☒	☐	Antibodies
☒	☐	Eukaryotic cell lines
☒	☐	Palaeontology
☒	☐	Animals and other organisms
☐	☒	Human research participants
☒	☐	Clinical data

Methods

n/a	Involved in the study	
☒	☐	ChIP-seq
☒	☐	Flow cytometry
☐	☒	MRI-based neuroimaging

Human research participants

Policy information about studies involving human research participants

Population characteristics	8 males and 6 females, ages 23-49, all with diagnoses of medication-intractable epilepsy
Recruitment	These were patients who were undergoing seizure monitoring for their medication-intractable epilepsy. All subjects were adequately consented. Systematic bias of epileptic patients possessing epileptiform discharges was controlled for by searching for and excluding these discharges from all analyzed electrodes. Thus, results are likely not impacted by subject recruitment. Subjects had a wide age range and were from both genders, so demographic bias does not exist.
Ethics oversight	This research was approved by the Columbia University Institutional Review Board under protocols AAAP4458 and AAAP5428

Note that full information on the approval of the study protocol must also be provided in the manuscript.

Magnetic resonance imaging

Experimental design

Design type	Anatomic monitoring
Design specifications	N/A. MRI images were obtained post-operatively for electrode localization
Behavioral performance measures	No behavioral performance measures were used relating to the MR images

Acquisition

Imaging type(s)	Structural
Field strength	3T
Sequence & imaging parameters	T2 coronal and whole-brain 3D T1
Area of acquisition	Medial Temporal Lobe

Diffusion MRI ☐ Used ☒ Not used

Preprocessing

Preprocessing software	Automatic Segmentation of Hippocampal Subfields multi atlas segmentation method was applied to pre-implantation high-resolution hippocampal coronal 3T T2-weighted and whole-brain 3D T1-weighted scans.
Normalization	Coregistration with post-op CT
Normalization template	Coregistration with post-op CT
Noise and artifact removal	Coregistration with post-op CT
Volume censoring	none

Statistical modeling & inference

Model type and settings	No statistical modeling or inference was done on the MR images.
Effect(s) tested	No effects were tested on the MR images.

Specify type of analysis: ☐ Whole brain ☒ ROI-based ☐ Both

Anatomical location(s): Medial Temporal Lobe

Statistic type for inference (See Eklund et al. 2016)	No statistic type was used for inference.
Correction	Coregistration with post-op CT

Models & analysis

n/a	Involved in the study
☒	☐ Functional and/or effective connectivity
☒	☐ Graph analysis
☒	☐ Multivariate modeling or predictive analysis
